# High-resolution anorectal manometry for diagnosing obstructed defecation syndrome associated with moderate rectocele compared to healthy individuals

**DOI:** 10.1186/s12876-023-03063-0

**Published:** 2024-01-04

**Authors:** Hong Zhi Geng, Yi Zhang, Chen Xu, Jiying Cong, Yuwei Li

**Affiliations:** 1 Department of Anorectal Surgery, Hepu People’s Hospital, Guangxi Zhuang Autonomous Region, 95 Dinghai North Road, Beihai City, 536100 China; 2 Departments of Colorectal Surgery, Tianjin Union Medical Center, 190 Jieyuan Road, Hongqiao District, Tianjin, 300121 China

**Keywords:** Rectocele, Obstructed defecation syndrome, Female, Anorectal manometry, Diagnosis

## Abstract

**Background:**

Few studies have investigated healthy female individuals (HFI) and those with obstructed defecation syndrome associated with moderate rectocele in women (MRW), identified using three-dimensional high-resolution anorectal manometry (3D HRAM) parameters that correlate with age stratification.

**Objective:**

We aimed to explore the clinical diagnostic values of the MRW and HFI groups using 3D HRAM parameters related to age stratification.

**Methods:**

A prospective non-randomized controlled trial involving 128 cases from the MRW (treatment group, 68 cases) and HFI (control group, 60 cases) groups was conducted using 3D HRAM parameters at Tianjin Union Medical Center between January 2017 and June 2022, and patients were divided into two subgroups based on their ages: the ≥50 and < 50 years subgroups.

**Results:**

Multivariate binary logistic regression analysis showed that age (*P* = 0.024) and rectoanal inhibitory reflex (*P* = 0.001) were independent factors affecting the disease in the MRW group. Compared to the HFI group, the receiver operating characteristic (ROC) curve demonstrated that the 3D HRAM parameters exhibited a higher diagnostic value for age (Youden index = 0.31), urge to defecate (Youden index = 0.24), and rectoanal pressure differential (Youden index = 0.21) in the MRW group.

**Conclusions:**

Compared to the HFI group, the ROC curve of the 3D HRAM parameters suggests that age, urge to defecate, and rectoanal pressure differential in the MRW group have a significant diagnostic value. Because the Youden index is lower, 3D HRAM cannot be considered the gold standard method for diagnosing MRW.

## Introduction

A rectocele in women is a hernia presenting itself as a bulge in the rectum wall to the side of the vagina, which may result in obstructed defecation syndrome (ODS) [1] due to a defect in the pelvic floor support structure or weakness in the function of the pelvic floor muscle. Consequently, patients have incomplete rectal emptying with or without a reduction in the number of bowel movements per week [2, 3].

Defecography is the first-line diagnostic method for assessing anorectal function and structural morphology [4]. It has revealed typical features of pelvic floor dysfunction, even in some asymptomatic individuals [5]. Our previous study showed differences in age and sex among healthy volunteers using three-dimensional high-resolution anorectal manometry (3D HRAM) [6]. Intrarectal pressure is related to age, body mass index (BMI), and parity [7], and the duration of sustained squeeze is shortened in multiparous females [8]. Age was also negatively correlated with balloon expulsion time (BET), whereas a higher BMI was associated with a higher maximum tolerated volume, longer BET, and greater length of the high-pressure anal zone (HPZ) in males than in females [9]. A study comparing the Eastern and Western literature found that the 3D HRAM parameters of healthy volunteers showed ethnic differences [10].

However, the accuracy of the diagnosis of defecation disorders using 3D HRAM has been questioned. Eighty-seven percent of healthy asymptomatic individuals and patients with dyssynergic defecation share similar 3D HRAM values [11]. The manometric parameters measured with the 3D HRAM probe cannot accurately predict prolonged BET [12], and normal anorectal manometry or BET results can occur in patients with dyssynergic defecation during defecography [13]. HRAM and magnetic resonance imaging explained only 36% of the variance in the bowel evacuation parameters, suggesting that these parameters did not fully capture the dynamic factors of the pelvic floor associated with successful defecation [14]. The clinical significance of normal and abnormal 3D HRAM values is unclear; however, when considering each functional parameter individually, individuals with parameter values outside the normal range may have no clinical symptoms, and patients with clinical problems may exhibit normal values. Given the enormous capacity of functional compensation, isolated dysfunction may not be clinically meaningful. Furthermore, the pathophysiology of most clinical diseases is multifactorial, and symptoms may appear only when multiple parameters are affected [15]. Therefore, no gold-standard diagnostic method exists for ODS [16].

Most studies have focused on the application of 3D HRAM in diagnosing dyssynergic defecation, which accounts for approximately 40% of ODS [17]. Few studies have explored the significance of 3D HRAM values in diagnosing ODS associated with moderate rectocele in women (MRW). Previous studies on normal 3D HRAM values in healthy volunteers stratified participants by sex, BMI, and age using different methods, and some studies did not stratify patients based on disease severity, which may have led to biased or opposite results [6, 9, 14]. More studies are urgently required to stratify patients according to age and disease severity to properly assess 3D HRAM accuracy in diagnosing ODS.

This study aimed to identify independent risk factors that influence the diagnostic value of the receiver operating characteristic (ROC) curve in the MRW and healthy female individual (HFI) groups by comparing the 3D HRAM parameters, which have practical clinical implications for the accurate diagnosis and stratified treatment of rectoceles.

## Materials and methods

### Study design and patients

Clinical data from patients diagnosed with ODS related to moderate rectocele by defecography between January 2017 and June 2022 at the Tianjin Union Medical Center were non-randomized and prospectively analyzed. 3D HRAM was performed in 90 patients with MRW as the treatment group to evaluate anorectal function before pelvic floor biofeedback therapy. Seventy HFIs were recruited as the control group and evaluated for 3D HRAM anorectal function. The age and BMI of the patients and individuals in the two enrollment groups were matched to reduce the effect of different baseline levels of these measures on diagnostic judgment, thus ensuring the precision of the results. After screening based on the inclusion and exclusion criteria, 68 patients and 60 individuals were included in the MRW and HFI groups, respectively. The included HFIs received 200 Yuan each for project funds. The Strengthening the Reporting of Observational Studies in Epidemiology statement [18] was followed in conducting this case-control study, and the study was approved by the ethics committee of Tianjin Union Medical Center (2022-B47). Moreover, this study complied with the tenets of the Declaration of Helsinki for medical research involving human individuals [19]. Written informed consent was obtained from all the patients and volunteers.

### Inclusion and exclusion criteria

#### Participant inclusion criteria

Females aged ≥18 years and <85 years; normal bowel movements within 6 months before enrollment: stool frequency <3 times/day and <3 times/week. The type of stool was Bristol 3–5, the duration of bowel movements was <10 min, and no strains of bowel movements, incomplete bowel movements, difficult or hand-assisted bowel movements, intestine-related movements, abdominal pain, intestine-related pain, or fecal incontinence were observed. Normal digital rectal examination results. No laxatives were administered for 3 months before enrollment.

#### Patient inclusion criteria

All patients were diagnosed according to the Rome IV diagnostic criteria for functional gastrointestinal disorders [20]. The moderate rectocele classification in Chinese defecography is as follows: rectocele diameter 16–30 mm [21]. For women aged ≥18 and < 85 years, defecography showing rectocele ≤30 mm and rectocele with perineal descent ≤35 mm [21].

#### Exclusion criteria

Pregnant women; patients with chronic systemic diseases, such as heart, lung, liver, and kidney disorders; patients with dyssynergic defecation, megacolon, and fecal incontinence identified by defecography or 3D HRAM; patients diagnosed with slow transit constipation using the slow transit test; patients with anorectal trauma (grade 3–4 lacerations) during delivery or before anorectal surgery, including hemorrhoid surgery; patients with psychotic disorders; or those who refused to cooperate.

### 3D HRAM studies

The patients received 80 ml of glycerin enema to clean their lower rectum before the 3D HRAM. The patients were placed in the left lateral decubitus position. A solid-state manometry assembly (ManoScanTM 3D; Sierra Scientific Instruments, Los Angeles, CA, USA, Factory number: 005) was used.

The 3D-HRM probe had the following characteristics: (1) the probe diameter was 10 mm, and the pressure-sensitive length of the probe was 64 mm. (2) The 3D HRAM probe comprised 256 sensors on the surface, which formed a continuous grid in the axial and circumferential directions, and one balloon reference sensor. (3) The spacing between the transducers was 4 mm in length in the axial direction or 2 mm in width in the circumferential direction.

The first and last calibration points were 0 and 300 mmHg, respectively. A solid probe was placed in the middle of the rectum, 10 cm from the anal edge. The 3D HRAM results were obtained by an investigator with 12 years of experience using a 3D HRAM probe. The alignment bump on the handle remained at the same reference angle as the patient throughout the procedure, regardless of position. Depending on the physician's choice, a tiny balloon catheter was introduced next to the manometry probe so that balloon inflation could be detected by a distal pressure transducer. The probe does not rotate or shift using a dedicated holder.

In our study, 3D HRAM parameters were evaluated according to the recommendations of the International Anorectal Physiology Working Group [22], as described in our previous study [6]. 3D HRAM equipment was used to measure the following indicators: rest (maximum resting pressure, mean resting pressure, and HPZ length), squeezing (maximum squeezing pressure and duration of sustained squeeze), simulated defecation (residual anal pressure, anal relaxation rate, intrarectal pressure, and recto-anal pressure differential), graded balloon distension (anorectal inhibitory reflex, first sensation, urge to defecate, and maximum tolerated volume), and defecation index.

HPZ was the length of the mean pressure curve in the resting pressure framework, defined as (rectal pressure + ([anal resting pressure-rectal pressure] ×0.25)) [9]. The eSleeve function identifies the maximum positive or minimum negative difference between the rectal and anal pressures during simulated defecation, measuring the rectoanal pressure differential = intrarectal pressure in 5 s of 20 s. During rectoanal inhibitory reflex (RAIR) assessment and simulated defecation, the anal relaxation rate (%) was calculated from the anal pressure measured within 1.5 s before the procedure. The anal relaxation rate (%) was calculated as 1− residual anal pressure/mean resting pressure ×100 [9]. RAIR was considered present if anal relaxation was > 25% [9]. Rectoanal pressure differential = intrarectal pressure−anal pressure [9]. Defecation index = intrarectal pressure/residual anal pressure [23]. All pressure measurements were performed with reference to atmospheric pressure, and with the exception of the rectal sensation phase, all formulas were automatically computed by the machine.

### Statistical methods

SPSS (version 22.0; SPSS Inc., Chicago, IL, USA) was used for statistical analysis. Categorical data are presented as numbers. Continuous data are presented as mean ± standard deviation. Devices and multivariate linear regression analysis were performed to test for the normal distribution of the data and homogeneity of the variance. If a heterogeneous distribution was found, the statistical method was changed. For normally distributed data, the independent sample t-test was used for intragroup comparisons, and the paired sample t-test was used for intergroup comparisons. Multivariate logistic regression analysis was used to screen for independent risk factors of MRW prognosis. The ROC curves were used to determine the diagnostic values of the two groups of parameters. *P* < 0.05 was considered statistically significant.

## Results

**Fig. 1:**
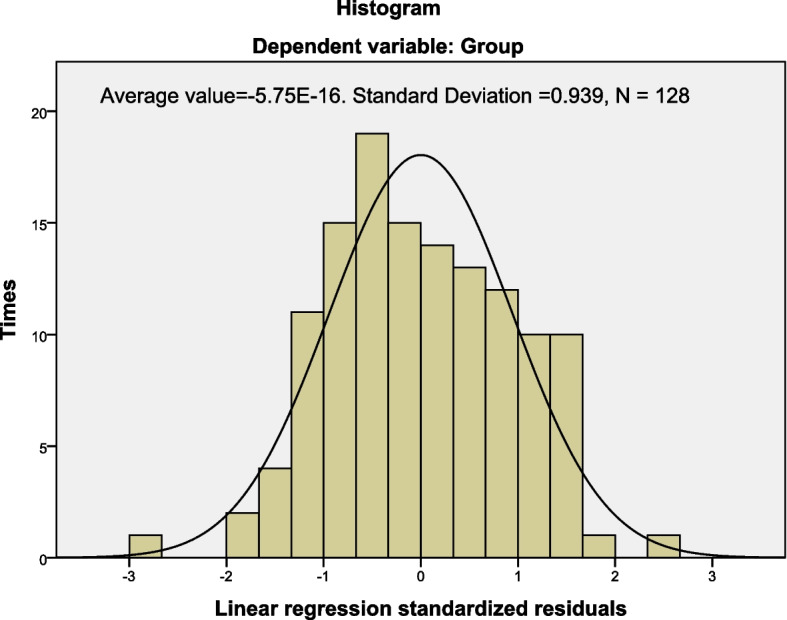
Histogram of multivariate linear regression of age, BMI and 3D HARM parameters in the MRW and HFI groups

**Fig. 2:**
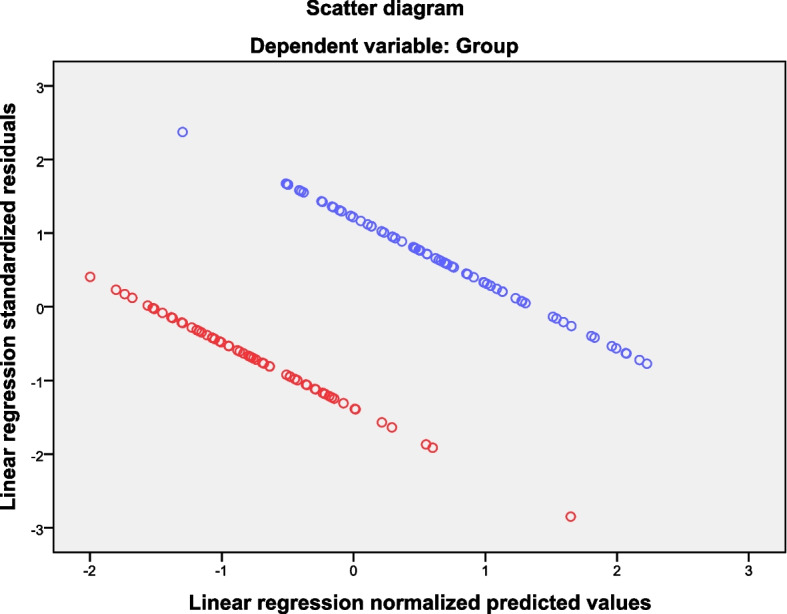
Scatter plots of multivariate linear regression of age, BMI and 3D HARM parameters in the MRW and HFI groups. Blue points indicate the MRW group, and red points refer to the HFI group

- 3.1 General data: HFI and MRW were identified using defecography in 128 patients. The age and BMI of participants in the HFI (60 cases) and MRW (68 cases) groups were not statistically significant (51.78 ± 12.29 vs. 52.94 ± 13.77, *P* = 0.92; 22.89 ± 4.09 vs. 22.93 ± 3.36, *P* = 0.97, respectively).
- 3.2 Normal distribution: The multivariate linear regression histogram showed that age and 3D HRAM parameters of participants in the HFI and MRW groups were normalized (Fig. 1) (F = 6.71, *P* = 0.00), indicating a good model fit. The scatter plots for the patients in the two groups were linearly independent, indicating homogeneity of the baseline data (Fig. 2).

- 3.3 In the HFI group, female age was negatively correlated with maximum resting pressure (*r* = - 0.28, *P* = 0.029) and maximum squeezing pressure (*r* = - 0.40, *P* = 0.001), whereas age was not correlated with 3D HRAM parameters in the MRW group.
- 3.4 The single factor analysis showed that maximum resting pressure (*P* = 0.00), mean resting pressure (*P* = 0.00), maximum squeezing pressure (*P* = 0.03), duration of sustained squeeze (*P* = 0.03), residual anal pressure (*P* = 0.00), intrarectal pressure (*P* = 0.02), rectoanal pressure differential (*P* = 0.01), and RAIR (*P* = 0.00) were lower in the HFI group compared to the MRW group (Table 1). Multivariate binary logistic regression analysis showed that age (*P* = 0.024) and RAIR (*P* = 0.001) were independent factors affecting the disease in the MRW group.

**Table 1:** Comparison of HFI and MRW groups in 3D HRAM parameters

Variable	HFI group(60 cases)	MRW group (68 cases)	HFI *vs.* MRW group	*P*
	Mean±SD	Mean±SD	*OR 95%*(*CI*)	
Rest				
Maximum resting pressure (mmHg)	69.71 ± 17.76	99.00 ± 22.71	4.18(-37.56~-21.03)	0.00
Mean resting pressure (mmHg)	61.60 ± 15.56	87.32 ± 23.36	3.56(-32.75~-18.67)	0.00
HPZ length (cm)	3.48 ± 0.65	3.71 ± 0.68	0.12(-0.46~0.00)	0.05
Squeeze				
Maximum squeezing pressure (mmHg)	169.14 ± 64.14	189.23 ± 49.88	9.24(-38.38~-1.81)	0.03
Duration of sustained squeeze (s)	14.46 ± 5.60	16.63 ± 5.35	0.97(-4.09~0.25)	0.03
Simulated defecation				
Residual anal pressure (mmHg)	64.27 ± 27.66	85.61 ± 31.59	5.28(-31.79~-10.89)	0.00
Anal relaxation rate (%)	26.80 ± 17.44	29.43 ± 28.29	4.22(-10.98~5.73)	0.54
Intrarectal pressure (mmHg)	41.12 ± 26.20	51.50 ± 22.42	4.30(-18.89~-1.88)	0.02
Rectoanal pressure differential (mmHg)	-19.40 ± 48.00	-38.10 ± 36.99	7.53(3.81~33.60)	0.01
Graded balloon distension				
Anorectal inhibitory reflex	16.17 ± 9.40	22.62 ± 9.15	1.64(-10.02~-3.52)	0.00
First sensation (cc)	38.17 ± 16.21	36.76 ± 19.66	3.21(-4.95~7.75)	0.66
Urge to defecate (cc)	88.33 ± 32.32	78.09 ± 36.74	6.15(-1.93~22.42)	0.10
Maximum tolerated volume (cc)	142.03 ± 35.03	136.91 ± 36.95	9.76(-28.82~9.99)	0.37
Defecation index	0.63 ± 0.62	0.68 ± 0.40	0.09(-0.30~0.07)	0.63

*HPZ* Anal high pressure zone, *3D HRAM* Three-dimensional high-resolution anorectal manometry, *HFI* Healthy female individuals. *MRW* Moderate rectocele in women

- 3.5 The individuals age ≥50 years subgroup for HFI showed lower maximum resting pressure (*P* = 0.00), mean resting pressure (*P* = 0.00), maximum squeeze pressure (*P* = 0.01), residual anal pressure (*P* = 0.00), intrarectal pressure (*P* = 0.02), rectoanal pressure differential (*P* = 0.03), and RAIR (*P* = 0.04) than the MRW subgroup. Individuals aged ≥50 years in the HFI subgroup had a significantly lower maximum squeezing pressure (*P* = 0.02) than those aged <50 years, and those in the MRW subgroup had a significantly lower anal relaxation rate (*P* = 0.03). Compared with individuals aged < 50 years in the MRW subgroup, those in the HFI subgroup had a significantly lower maximum resting pressure (*P* = 0.03), mean resting pressure (*P* = 0.03), and RAIR (*P* = 0.00) (Table 2).

**Table 2:** Comparison of 3D HRAM parameters between the HFI and MRW subgroups at different ages

Variable	Individuals aged ≥ 50 years (49 cases) *vs.* < 50 years (*n* = 11) in the HFI subgroup	*P*	Individuals aged ≥ 50 years (47 cases) vs. < 50 years (*n* = 21) in the MRW subgroup	*P*	Individuals aged ≥50 years HFI group (49 cases) *vs.* ≥50 years MRW subgroup (47 cases)	*P*	Individuals aged < 50 years HFI subgroup (11 cases) *vs.* < 50 years MRW subgroup (21 cases)	*P*
	*OR 95%*(*CI*		*OR 95%*(*CI*)		*OR 95%*(*CI*)		*OR 95%*(*CI*)	
Rest								
Maximum resting pressure (mmHg)	5.90(-19.10~4.52)	0.22	7.31(-10.49~18.70)	0.57	4.93(-41.82~-22.24)	0.00	8.31(-37.37~-3.46)	0.03
Mean resting pressure (mmHg)	5.17(-16.44~4.27)	0.24	6.14(-6.70~17.82)	0.37	4.10(-36.69~20.37)	0.00	7.95(-32.73~0.17)	0.03
HPZ length (cm)	0.22(-0.47~0.40)	0.87	0.18(-0.54~0.17)	0.30	0.14(-0.47~0.08)	0.16	0.25(-0.81~0.22)	0.26
Squeeze								
Maximum squeezing pressure (mmHg)	17.51(-77.64~-7.52)	0.02	13.18(-30.19~22.45)	0.77	9.49(-45.64~--7.94)	0.01	25.55(-40.71~63.97)	0.61
Duration of sustained squeeze (s)	1.88(-4.84~2.69)	0.57	1.41(-3.27~2.38)	0.76	1.15(-4.43~0.12)	0.06	2.03(-5.89~0.15)	0.38
Simulated defecation								
Residual anal pressure (mmHg)	9.29(-22.73~14.47)	0.66	8.29(-7.81~25.28)	0.30	6.03(-36.69~-12.73)	0.00	11.60(-36.18~11.13)	0.29
Anal relaxation rate (%)	5.79(-19.07~4.10)	0.20	7.21(-30.53~-1.72)	0.03	4.52(-7.71~10.44)	0.77	9.59(-27.29~-11.84)	0.43
Intrarectal pressure (mmHg)	8.80(-21.47~13.76)	0.39	5.90(-7.02~16.54)	0.42	5.13(-23.24~-2.24)	0.02	7.27(-18.61~11.06)	0.61
Rectoanal pressure differential (mmHg)	16.14(-27.42~37.20)	0.76	9.78(-20.89~81.17)	0.89	9.09(1.77~37.86)	0.03	14.09(-14.48~43.01)	0.32
Graded balloon distension								
Anorectal inhibitory reflex	3.13(-3.18~9.38)	0.33	2.42(-6.08~3.1807)	0.61	1.97(-9.79~-1.96)	0.04	3.09(-14.64~2.30)	0.00
First sensation (cc)	5.44(-7.56~14.20)	0.54	5.20(-8.85~11.89)	0.77	3.76(-6.30~8.63)	0.76	6.64(-13.09~14.00)	0.95
Urge to defecate (cc)	5.44(-7.56~14.20)	0.25	9.71(-17.34~21.45)	0.83	7.39(-3.63~25.72)	0.14	12.24(-23.20~26.93)	0.78
Maximum tolerated volume (cc)	10.75(-17.75~29.45)	0.62	9.77(-17.08~21.92)	0.81	7.18(-9.30~19.33)	0.49	14.72(-25.48~34.57)	0.76
Defecation index	0.21(-0.71~0.12)	0.16	0.10(-0.17~0.25)	0.96	0.11(-0.34~0.11)	0.28	0.14(-0.06~0.51)	0.12

*HPZ* Anal high pressure zone, *3D HRAM* Three-dimensional high-resolution anorectal manometry, *HFI* Healthy female individuals, *MRW* Moderate rectocele in women

**Fig. 3:**
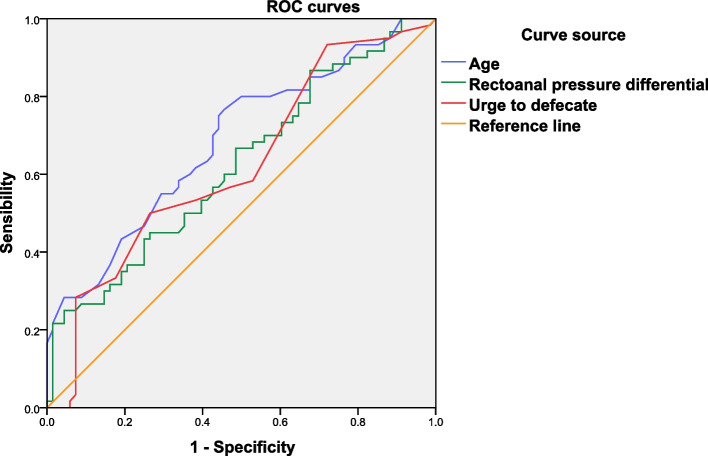
Compared to the HFI group, the ROC curve demonstrated that 3D HRAM parameters had a higher diagnostic value for age > urge to defecate > rectoanal pressure differential in the MRW group, as indicated by a superior Youden index

- 3.6 Diagnostic value of 3D HRAM parameters in the HFI group compared with the MRW group. In the MRW group, the cutoff point for the decline of anal function was at 53.5 years of age, with a sensitivity of 77%, a specificity of 54%, a Youden index of 0.31, and an area under the curve (AUC) of 69% (*P* = 0.00). When the rectal fecal volume reached 95 cc, the MRW group exhibited a cutoff point for patients who had the urge to defecate, with a sensitivity of 50%, a specificity of 74%, a Youden index of 0.24, and an AUC of 62% (*P* = 0.16). When the rectoanal pressure difference reached -1.35 mmHg, the MRW group exhibited a cutoff point for patients who had the first sensation, with a sensitivity of 25%, specificity of 96%, Youden index of 0.21, and an AUC of 62% (*P* = 0.16). Compared to the HFI group, the ROC curve demonstrated that the 3D HRAM parameters exhibited a higher diagnostic value for age, urge to defecate, and rectoanal pressure differential in the MRW group, as evidenced by the higher Youden index (Table 3, Fig. 3).

**Table 3:** The ROC curves demonstrate the diagnostic value of the 3D HRAM parameters for the MRW group compared to the HFI group

Parameters	Cutoff point	Sensitivity	Specificity	Youden	AUC	*P*
Age	53.5(years)	77%	54%	0.31	69%	0.00
Urge to defecate	95(cc)	50%	74%	0.24	62%	0.16
Rectoanal pressure differential	-1.35(mmHg)	25%	96%	0.21	62%	0.16

*HFI* Healthy female individuals, *MRW* Moderate rectocele in women

**Fig. 4:**
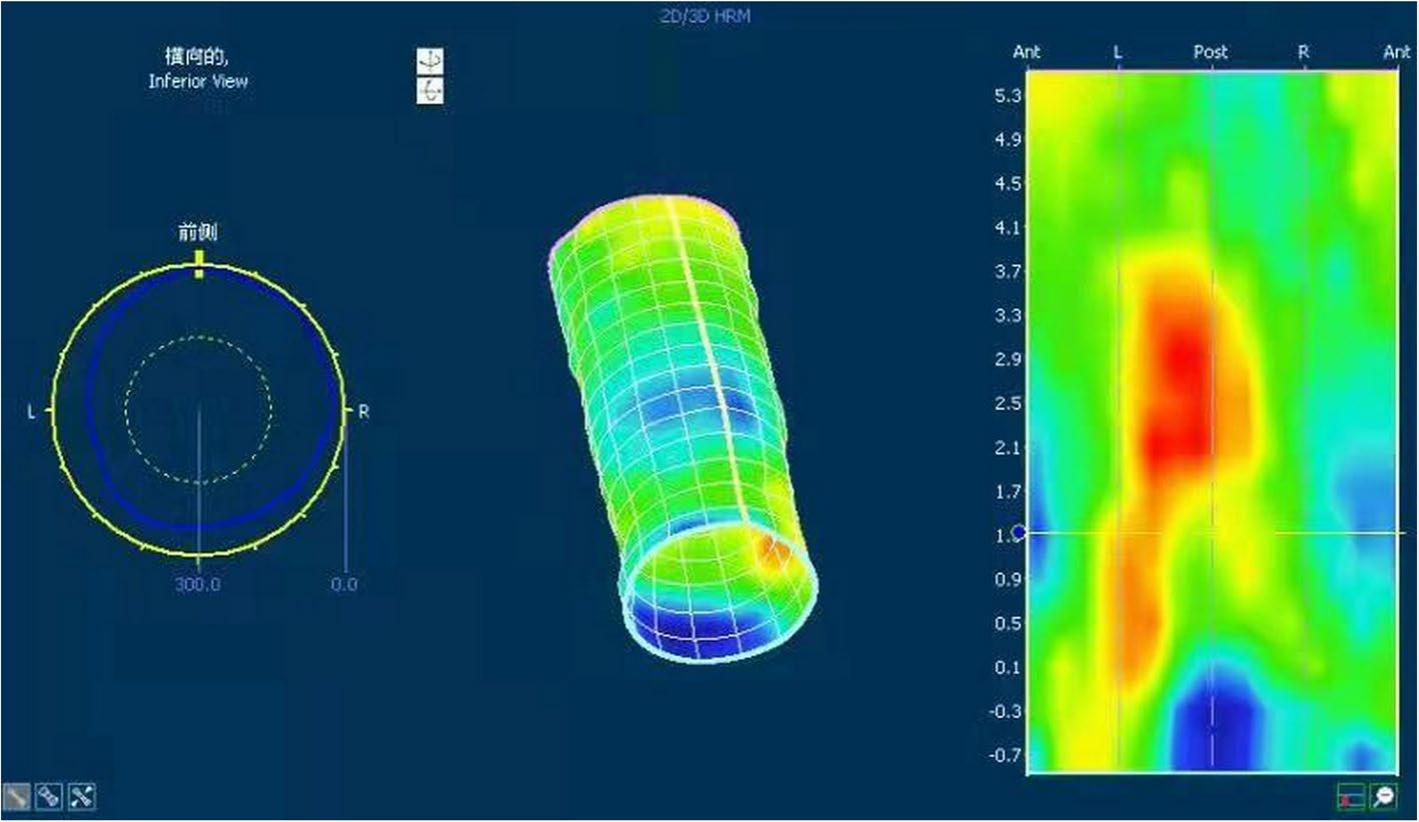
The 3D HRAM map of MRW shows characteristic low-pressure areas in blue. 3D HRAM = three-dimensional high-resolution anorectal manometry. HFI = healthy female individuals. MRW = moderate rectocele in women

- 3.7 The 3D HRAM map of MRW shows characteristic low-pressure areas in blue (Fig. 4).

## Discussion

In this study, 3D HRAM was used to assess the diagnostic value of age and disease severity stratification with respect to HFI and MRW. The biggest advantage of 3D HRAM is that it can be used for two-dimensional (2D) and 3D analysis. The color contour map can be used to understand the pressure curve more intuitively; the 3D map shows the blue low-pressure band of the characteristic areas of rectal defects in patients with MRW (Fig. 4). However, the accuracy of 3D HRAM diagnosis was affected by the technician’s level of operation, and the 3D HRAM operator in this study had 12 years of experience. Therefore, the pressure measurement parameters were normally distributed with a uniform variance, indicating that our parameters were reliable and usable.

In this study, a cutoff point method for age ≥50 years was adopted as in previous studies [8, 10, 24–26]. Comparing the ≥ 50-year to <50-year subgroup, the maximum squeezing pressure was lower in the HFI group. Multivariate binary logistic regression analysis showed that age was an independent risk factor of MRW. In the HFI group, a negative correlation was found between age, maximum resting pressure, and maximum squeezing pressure, but not in the MRW group. Li et al. reported a negative correlation between age and the maximum resting, mean resting, and maximum squeezing pressures in the HFI group [6]. Noelting et al. reported that age >50 years was associated with lower resting anal pressure in the HFI group; however, age < 50 years was not associated with squeezing pressure, and younger females had a more negative rectal pressure differential than older females [23]. Coss-Adame et al. reported a significant reduction in resting and sustained squeeze pressures in women aged > 50 years [25]. The differences in these results may be related to the differences in age, parity, BMI, ethnicity, and grouping methods of the volunteers enrolled in the study.

The difference in these 3D HRAM parameters between the MRW and HFI groups in this study may be related to the prolapse of the rectal mucosa and fecal matter within the rectal thrust-forward congestion of the anus. RAIR sensitivity was lower than that of HFI, resulting in an increase in the maximum resting and mean resting pressures. To facilitate smooth defecation, the residual anal, intrarectal, and rectoanal pressure differential increased; simultaneously, the maximum squeezing pressure was increased to maintain continence. Prichard et al. reported that intrarectal pressure, perineal descent scores during excretion, and dilated anal canals were associated with rectoceles > 3 cm [14]. This is consistent with our findings: age was not associated with MRW. However, this study reported that the age of the HFI group was negatively correlated with the maximum resting and squeezing pressures. Patients with abnormal rectal structure have lower median intrarectal pressure and a negative median rectal gradient during excretion [14]; however, a BET subgroup analysis was not performed. The mean resting pressure is the pressure at which the anorectal resting level reflects the function of the internal anal sphincter, and a high anal resting pressure may indicate smooth or striated muscle spasms [14]. The maximum squeezing pressure and duration of sustained squeeze reflect the function of the external anal sphincter and pelvic floor muscles, while the duration of sustained squeeze is a manifestation of the endurance of the slow muscle, with females squeezing less than males and older people squeezing less than younger people [6–9, 24, 25]. Coss-Adame et al. reported a significant reduction in rest and duration of sustained contractions in females aged > 50 years [25]. Furthermore, differences in residual anal, intrarectal, and anorectal pressures reflect the propulsive force exerted on the anorectum during simulated defecation.

The RAIR is an essential component of normal defecation and is characterized by decreased intrarectal pressure during rectal balloon distention. RAIR is characterized by relaxation along the anteroposterior axis, length of the canal, and differences with the vector, with the largest changes occurring at the internal anal sphincter level [26], and the magnitude and duration of RAIR depend on the rate and volume of rectal dilation [27]. A non-HRAM study detected RAIR deletions with a diagnostic utility of 91% and a specificity of 94% [28]. Neural pathways and reflex mechanisms in the muscles of the colon, rectum, anal sphincter, and pelvic floor are involved in defecation and continence [29]. The defecation receptor is located in the rectum, and RAIR reflects the sensitivity of the rectal defecation receptor in the brain and the reflex arc of rectal defecation [30]. When RAIR is initiated, a large amount of rectal content enters the lower rectum, and the sympathetically driven defecation reflex begins unless inhibition is consciously felt [31]. Multivariate binary logistic regression analysis showed that age and RAIR were independent factors affecting the disease.

The Youden index, also known as the correct index, refers to the sum of sensitivity and specificity minus one; thus, the range of index values is 0–1, and the larger the Youden index, the better the authenticity. The ROC curve demonstrated that the 3D HRAM parameters exhibited a higher diagnostic value for age, urge to defecate, and rectoanal pressure differential in the MRW group, as evidenced by the superior Youden index. Because the Youden index is low, 3D HRAM cannot be used as the gold standard for diagnosing MRW.

Our study was a prospective non-randomized controlled trial with potential weaknesses, including a small sample size after subgroup analysis. Moreover, we did not have data on the number of patients with hand-assisted bowel movements. The prevalence of sphincter defects was higher in females who delivered vaginally and increased with parity [32]. However, the number of females who delivered vaginally and the normal and abnormal BET for both groups were not statistically recorded. Furthermore, more randomized controlled trials are needed to investigate the effect of the simulated defecation squatting and left lateral decubitus positions on 3D HRAM parameters. A control group for MRI defecography and 3D ultrasound is required to determine the diagnostic value of 3D HRAM for MRW.

In this study, age and RAIR were screened as independent risk factors for MRW using 3D HRAM parameters from binary logistic regression analysis. Compared to the HFI group, the ROC curve of the 3D HRAM parameters suggests that age, urge to defecate, and rectoanal pressure differential in the MRW group have significant diagnostic value. Because the Youden index was lower, 3D HRAM cannot be considered a gold standard for diagnosing MRW. Our findings are of practical interest for the accurate diagnosis and stratified treatment of rectocele.

## Data Availability

The datasets generated and/or analyzed during the current study are not publicly available but are available from the corresponding author upon reasonable request.
